# Supplementary material for: Splice-Junction-Based Mapping of Alternative Isoforms in the Human Proteome
Source: Cell Rep. Author manuscript; Available in PMC 2020 Jan 15. (PMC6961840; doi:10.1016/j.celrep.2019.11.026)
Supplement: 2 [file NIHMS1546469-supplement-2.zip › DF1/PXD010154/Ovary/Ovary_3_DRAP1_VAVEEGLPGDPVAER.pdf]

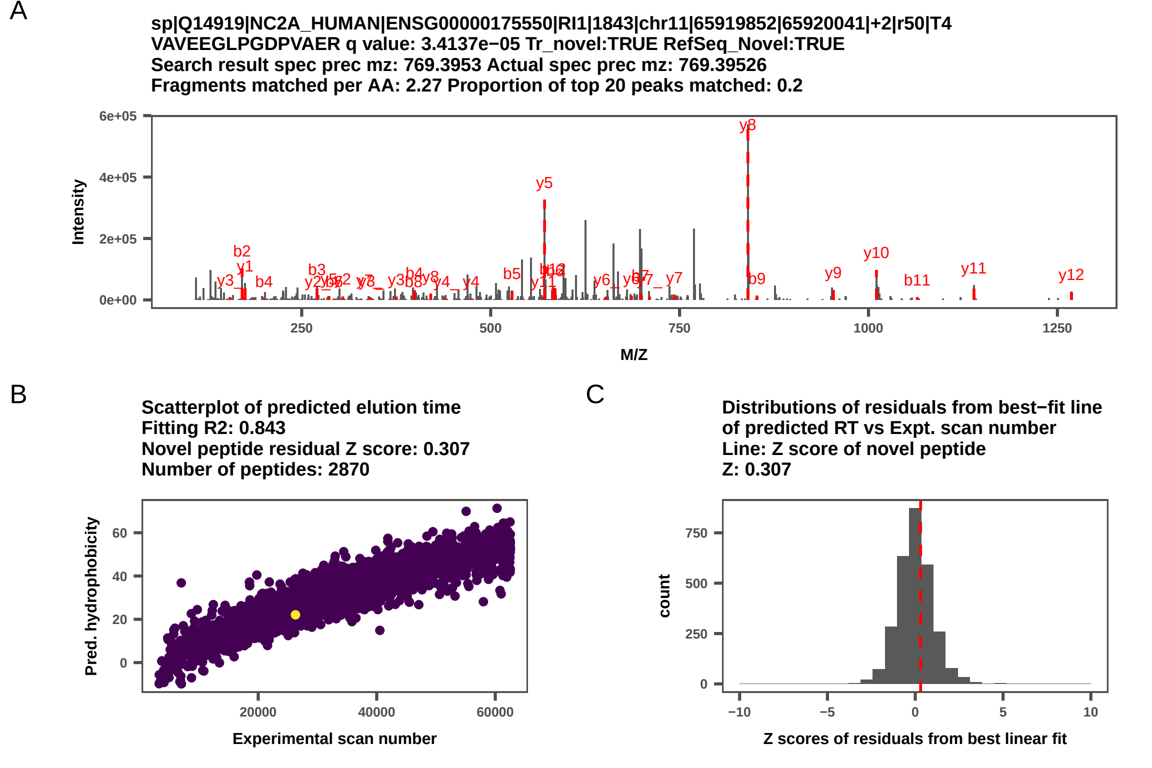
A
sp|Q14919|NC2A_HUMAN|ENSG00000175550|RI1|1843|chr11|65919852|65920041|+2|r50|T4
VAVEEGLPGDPVAER q value: 3.4137e−05 Tr_novel:TRUE RefSeq_Novel:TRUE
Search result spec prec mz: 769.3953 Actual spec prec mz: 769.39526
Fragments matched per AA: 2.27 Proportion of top 20 peaks matched: 0.2
Intensity
6e+05
4e+05
2e+05
0e+00
y8
y5
b2
y1
y3
b4
b3
y10
y9
y11
y12
b11
b9
b5
y6
y7
250
500
750
1000
1250
M/Z
B
Scatterplot of predicted elution time
Fitting R2: 0.843
Novel peptide residual Z score: 0.307
Number of peptides: 2870
Pred. hydrophobicity
60
40
20
0
20000
40000
60000
Experimental scan number
C
Distributions of residuals from best−fit line
of predicted RT vs Expt. scan number
Line: Z score of novel peptide
Z: 0.307
count
750
500
250
0
−10
−5
0
5
10
Z scores of residuals from best linear fit
